# Diseases of Respiratory Organs

**Published:** 1905-10-14

**Authors:** 


### DISEASES OF RESPIRATORY ORGANS.

(*Continued from page* 9.)

**The Diazo Test.**—Apart from the value of this test in typhoid fever it is desirable to discover whether it is fairly constant in tuberculosis and what indications its presence affords as to the future progress of the case. Ch. W. Budden[6] has investigated the reaction in 3,000 cases, 600 of of them being persons in good health, 672 tuberculous, and the remainder affected with various diseases. Many of them were tested daily and the inquiry seems to have been threshed out thoroughly at least as to tuberculosis. The results are somewhat disappointing. The reaction however does not occur with any disease except these five: measles, typhus, typhoid, puerperal fever, and tuberculosis. When it is found during bronchitis or pneumonia or other diseases it is probably due to an underlying tuberculosis, and so far it is a useful sign. The absence of the reaction is, on the other hand, of little value. Even in tuberculosis it can never be depended on. It disappears for a considerable period in some patients and then reappears. In early cases it is generally absent, nor does Budden find that it is of any value for prognosis in chronic phthisis. The positive reaction usually occurs among bad cases, but some of these run a very slow course, and others a rapid one. The reaction may disappear either because the patient is improving or growing weaker, and it vanishes altogether before death. On the whole, then, it seems that if the test be accurately carried out a positive reaction shows the presence of one of five diseases, and if we can exclude all but tuberculosis, this is of a severe type, or in an active stage. Such cases, however, usually present other indisputable symptoms.

**Prevention of Tuberculosis.**—The Henry Phipps Institute in the United States has been created to take a most important part in the campaign against tuberculosis. In its first annual report an account is given of the occupation and condition of the patients in the special hospital.[7] Of the 851 women the most numerous classes were house-workers, factory hands, weavers, and seamstresses; while among the men were labourers, tailors, clerks, and cigar-makers, alcohol vendors, mill hands, machinists, and weavers. Exposure to contagion appeared to be perhaps the most important factor in the spread of the disease. The right lung seemed to be most often affected first. Of 1,460 cases 148 had suffered for more than ten years and 171 for five. Their dispensary patients are drilled in preventative measures, and visited periodically at home by a nurse to aid them in carrying out the teaching, while other patients are taken into the hospital itself. The importance of such work in large cities, in addition to the formation of sanatoria, is emphasised in the address of Dr. Arthur Latham.[8] He refers to the need of cheap dispensaries where the sputa could be tested to aid in the early recognition of the disease, and the linking up of all public hospitals and similar institutions so that a patient could be quickly sent to the special hospital suited to his case. There is also need for the support of families when the breadwinners are in sanatoria, and for the patients some time after dismissal. Bernheim,[9] in an investigation of the causes of the spread of tuberculosis, also comes to the conclusion that the insanitary house, and especially the previously infected one, is the chief cause. The disease is almost unknown among savages and found chiefly in cities. Statistics show it varies with the poverty and overcrowding of the people, and particularly with the presence of dwellings in which previous cases have lived. The mortality has fallen where, as in England, general sanitary improvement has been marked, apart from special measures directed against the disease. Hence he argues for the sanitary inspection of all lodgings and the compulsory disinfection of all rooms where tuberculous patients have lived.

**Rest for the Consumptive.**—Dr. T. H. Hughes[10] points out that, next to pure air and copious food, rest is the most important need of the phthisical, and that it is the failure to obtain complete rest which is the cause of so few patients being cured at home. Thorowgood,[11] too, emphasises the danger of allowing patients when convalescent to go tours with healthy persons if the amount of exercise is not strictly limited; while Woods Hutchinson,[12] arguing in favour of the most complete rest for the lung and for the individual, endeavours to find a physiological basis for his argument in the researches of Robin and Binet. These authors showed that the total air expired by consumptives is 80 per cent. greater than that poured out by normal individuals, the oxygen consumed is 70 per cent., and the $CO_2$ exhaled is 64 per cent. greater. In other words, the consumptive is one whose respiratory processes are excessive. Finally, they discovered that the only method by which they could reduce this excessive gaseous interchange was by keeping the patient absolutely at rest. This tendency to excessive metabolism is found even in the pre-tubercular state, before the actual development of the disease. The success of over-feeding in treatment and the use of fatty food, cod-liver oil, and creasote all point to the value of anything which hinders this excessive metabolism. Physical and mental rest is not the least of our means of meeting the end thus indicated, but is too often overlooked in our anxiety to give an open-air life.

[6] Brit. Med. Jour., May 6. [7] Med. Record, March 18. [8] Lancet May 6. [9] Med. Record, April 8. [10] Brit. Med. Jour., April 22. [11] Brit. Med. Jour., May 20. [12] Med. Record, April 29.

